# Measurement of dose‐area product with GafChromic XR Type R film

**DOI:** 10.1120/jacmp.v6i3.2047

**Published:** 2005-08-17

**Authors:** George Thomas, Yushan Li, Robert Y.L. Chu, John Y. Cheung, Feroz Maqbool, Frank Rabe, G. Scott Burns

**Affiliations:** ^1^ Department of Radiological Sciences University of Oklahoma Health Science Center 800 Northeast Thirteenth St. Oklahoma City Oklahoma 73104; ^2^ Department of Electrical and Computer Engineering University of Oklahoma 1610 Asp Ave. Norman Oklahoma 73072; ^3^ Radiology Service Veterans Affairs Medical Center 921 Northeast Thirteenth St. Oklahoma City Oklahoma 73104; ^4^ Department of Radiology Hillcrest Medical Center 1120 South Utica Ave. Tulsa Oklahoma 74104; ^5^ Cancer Management Services Norman Regional Hospital 901 N. Porter Norman Oklahoma 73071 U.S.A.

**Keywords:** GafChromic film, scanner, densitometer, dose‐area product, DAP

## Abstract

Many of the newer X‐ray machines are equipped with electronic means to provide dose–area product (DAP) information. For machines without that ability, an alternative method is to record radiation on a film that can handle a large amount of cumulative exposure. The use of GafChromic XR Type R film was investigated for this purpose by placing it at the X‐ray tube assembly to record the radiation in interventional radiological procedures. Dose‐area product was determined with a reflective densitometer and then with a flatbed scanner. Precisions were demonstrated to be 5% and 2%, respectively. In a comparison with the machine‐recorded DAP, a regression analysis showed the validity of both techniques for values less than 1200 Gy‐cm^2^.

PACS numbers: 87.52.Df, 87.66.Cd

## I. INTRODUCTION

Increasing concern for the potentially high radiation dose in fluoroscopy‐guided interventional procedures has led to the use of a variety of techniques to monitor patient dose.^(^ ^1^ ^–^ ^6^ ^)^ Many X‐ray machines of recent design are able to provide information on radiation exposure in the form of dose‐area product (DAP). An example device would be a large‐area ionization chamber installed in the X‐ray machine, which would allow radiation output to be measured directly. For X‐ray machines lacking similar capability, it would be desirable to find an alternative means to measure radiation output. A relatively new type of film, the GafChromic XR Type R Dosimetry film (International Specialty Products, Wayne, NJ), was introduced to record skin entrance radiation exposure in interventional radiological procedures. Its ability to record high radiation dose has been demonstrated.^(^ ^7^ ^,^ ^8^ ^)^ The GafChromic XR Type R film could therefore be an alternative means to record DAP. Such a possibility was investigated here by placing the film at the output of an X‐ray tube assembly and then measuring the film response by a handheld reflective‐type densitometer and an economical flatbed scanner.

## II. METHODS

Two types of interventional radiological procedures were used in this investigation: endograft and arch four‐vessel arteriogram. The former would give very high radiation exposure, whereas the latter would give a moderate amount. In a total of 17 cases (4 endografts and 13 arteriograms), DAP was determined as described later in this section. All cases, except one, were done using the Philips Optimus V5000 stationary X‐ray system (Philips Medical Systems, Andover, MA). The exception was done with a portable Philips BV Endura fluoroscopic/radiographic unit. For each patient, the cumulative DAP displayed by the X‐ray machine was recorded. In addition, the radiation was recorded by a GafChromic XR Type R film, which covered the collimator aperture. Dose–area product from the film was obtained by two methods: “manually” with a densitometer and “automatically” with a scanner.

### A. X‐ray systems

High‐voltage calibration of the Philips Integris V5000 X‐ray system was checked with a Radcal noninvasive kilovoltage detector, Model 4081 (Radcal Corporation, Monrovia, CA), at six points between 69 kVp and 108 kVp. The half‐value layer of the system was determined at 85 kVp, with a Radcal Radiation Monitor, Model 1575, with thimble‐size ion chamber, model 10x‐5‐6, for the fluoroscopic mode. The dependence of displayed DAP on the kilovoltage was determined by comparing the ratio of the DAP to thimble ion chamber measurement over a range of tube voltages (64 kVp to 106 kVp). Finally, calibration of the displayed DAP was performed by a comparison to the product of the measurement by the thimble ion chamber and the irradiated area recorded by a film (X‐OMAT TL, Eastman Kodak Company, Rochester, NY). The Philips BV Endura portable system's beam quality was similarly determined by the measurement of kilovoltage and half‐value layer. The displayed DAP value was also calibrated as described above.

### B. Using the densitometer to calculate the dose‐area product

**Figure 1:**
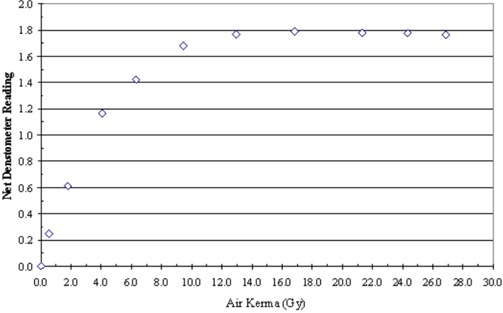
Film response as measured by a reflective densitometer

**Figure 2:**
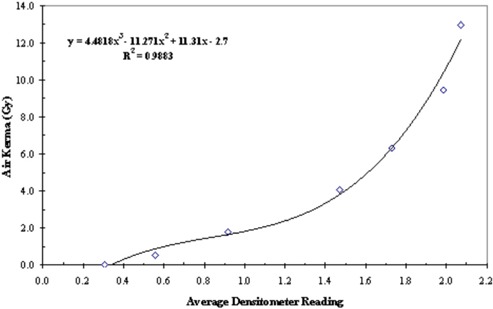
Calibration using densitometer's measurement of a reference pattern. Solid line is a fit by a polynomial function of the third order.

The response of the film to the radiation was measured with a modified reflective‐type densitometer, the Tobias RPB densitometer (Tobias Associates, Ivyland, PA).^(^ ^8^ ^)^ The conversion of the measurements to air kerma and the correction of the DAP are described below. The creation of a step wedge‐like reference pattern has been described in an earlier publication.^(^ ^8^ ^)^ Pieces of the GafChromic film were exposed to different, but known, amounts of radiation from the Philips V5000 X‐ray system at 75 kVP. The radiation exposure was converted to air kerma (in grays). A plot of the film response in terms of net reflection density versus air kerma established the limiting response to be at about 14 Gy (Fig. 1). A reference pattern within this limit was made for each batch of films purchased. A densitometer was used to read the optical density from each step in the reference pattern. Three readings were taken at each step, and an average value was computed. A polynomial regression analysis to the third order was used to graph average densitometer reading versus air kerma (in grays). An example is presented in Fig. 2.

**Figure 3:**
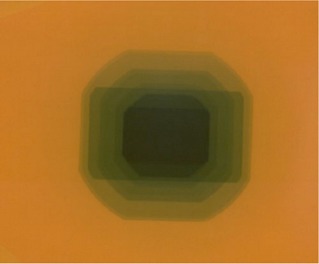
Film after irradiation at the X‐ray tube assembly of a stationary machine

Every GafChromic film irradiated at the X‐ray tube assembly displayed distinct regions of different amounts of radiation exposure (Fig. 3). Five points from each region were measured by the Tobias Associates RPB densitometer. The average density was calculated. Using the relation of density to air kerma derived from the corresponding calibration tablet, this mean value was converted to air kerma (in grays). A sheet of linear graph paper was laid over the film, and the outlines of all the regions were traced. Areas were estimated by counting the squares enclosed by the outlines. These areas were multiplied by the respective mean air kerma value. The sum of these results would be the DAP obtained manually.

Both the intraobserver variability and interobserver variability of the manual techniques were assessed as follows. One image (Fig. 3) was chosen, and the areas were measured five times by one operator to produce five DAP values. The mean and standard deviation were computed. Then five different people measured areas in the same image to produce five different DAP values. Again, the mean and standard deviation were computed.

### C. Using a scanner to calculate the dose‐area product

**Figure 4:**
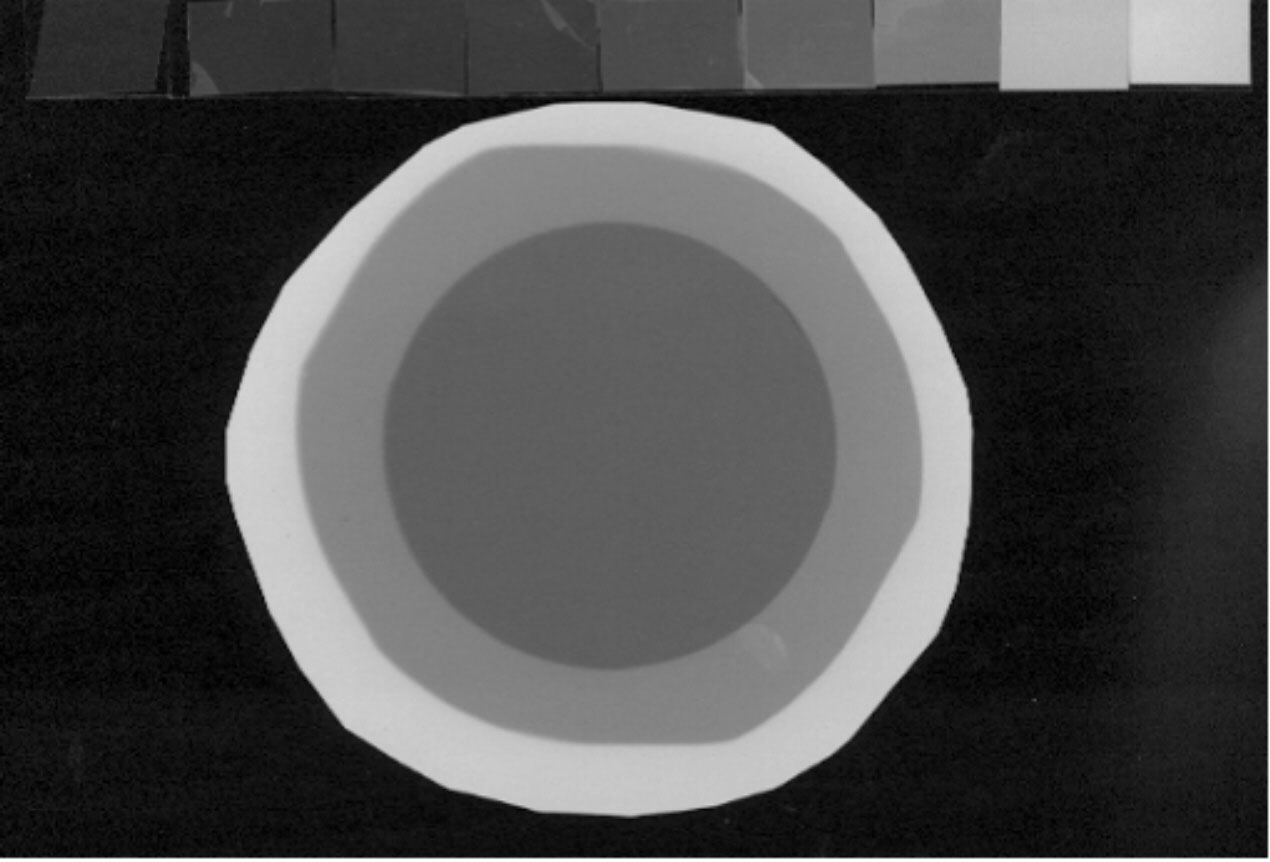
Red component of the recorded image of radiation output at the X‐ray tube assembly of a portable machine (bottom) and the corresponding calibration pattern (top)

Each film placed at the X‐ray tube assembly was scanned with a Microtek ScanMaker 4800 (Microtek USA, Carson, CA) with the technical protocol established in the previous investigation.^(^ ^8^ ^)^ (The scanning parameters were as follows: RGB mode, 300 dpi, and 25% scaling of the image. No color correction factors or filters were used.) Each image was scanned with the corresponding reference pattern and then split into color components by Paint Shop Pro 7.0 (Jasc Software, Eden Prairie, MN). The red component (Fig. 4) was then processed by MATLAB (The MathWorks, Inc. Natick, MA) as follows.

**Figure 5:**
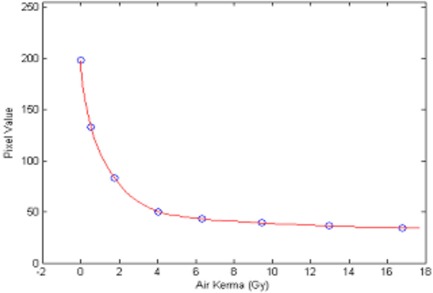
Film response as measured by a flatbed reflective scanner. Solid line is a fit by a piecewise polynomial of the third order.

**Figure 6:**
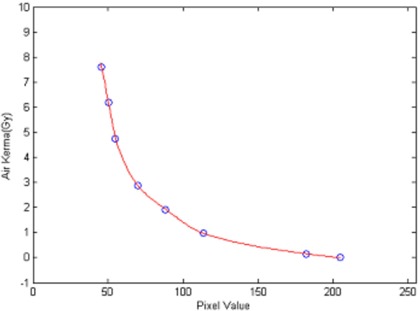
Calibration using scanner's measurement of reference pattern. Solid line is a fit by a piecewise polynomial of the third order.

The step wedge‐like reference pattern used in the previous section was also used here to establish the limiting response of the film. A plot of the film response in terms of pixel value versus air kerma determined the limiting response to be at about 6 Gy (Fig. 5). A reference pattern within this limit was made for each batch of films purchased. Calibration was performed by analyzing this reference pattern. An area of interest (55 pixels by 35 pixels) was placed inside a step, and the mean pixel value was computed. The relation of mean pixel values of the steps to the respective known air kerma values was fitted by a piecewise polynomial interpolation method as provided by the MATLAB software. An example is presented in Fig. 6.

The image of the radiation output at the X‐ray tube assembly might contain a few aberrant pixels (much less than 1% of the total number of pixels in the area of interest). These pixels had values significantly greater or lesser than their neighbors and were located in the portion of the film with the highest radiation exposure. So each image was filtered by comparing each pixel to its immediate neighbors. If the center pixel value was found to be larger or smaller than the surrounding pixels by more than three standard deviations, that pixel value was discarded and replaced by the value of the previous pixel. Each pixel in the filtered image was then converted to air kerma by piecewise polynomial interpolation (Fig. 6). The area of each pixel can be obtained by the whole image area divided by total pixels. The product of the sum of air kerma values of all pixels and the area of a pixel would then be the DAP.

The intraobserver variability and interobserver variability were assessed in the following manner. One film was scanned five times in one day without any change in the position of the film in the scanner. For each image, the DAP was computed as described above. The mean and standard deviation of the DAP values were calculated. The same film was also scanned in five different locations in the scanner. Again, the DAP was computed for each image, and the mean and standard deviation of the five DAP values were calculated.

## III. RESULTS

### A. Calibration of the dose–area product meter

For the Philips Integris V5000 X‐ray system, the comparison between the indicated and measured kilovoltage had a maximum difference of 7.8%. For the range of 70 kVp to 90 kVp used for the patient procedures, the difference was less than 5.0%. The half‐value layer was determined to be 5.28 mm of Al at 84 kVp. There might be a slight decrease in the DAP‐to‐ion chamber ratio as the kilovoltage increased from 64 kVp to 106 kVp. The change would be less than the 4.00% uncertainty in the calibration of the thimble ion chamber (k=2 at a 95% confidence level). Relative to the DAP determined by the thimble ion chamber, the machine‐indicated DAP value slightly overestimated the measured value by a factor of 1.06 at 76 kVp. All recorded DAP values in this investigation were corrected by this factor.

For the Philips BV Endura portable system, the comparison between the indicated and measured kilovoltage has a maximum difference of 3.7%, and this is at the usual kilovoltage range (65 kVp to 80 kVp) for these types of cases. The half‐value layer was determined to be 5.40 mm of Al at 80 kVp. Again, the displayed DAP appeared to have a dependence on kilovoltage. As the kilovoltage increases, the DAP‐to‐ion chamber ratio decreases. There is a change of 11.0% from 60 kVp to 100 kVp. The indicated DAP value was found to be larger than the measured value by a factor of 4.40. As a result, there is a grossly overestimated display value for the case done with this machine. This overestimated value can be corrected by the factors discussed above and therefore make this particular study still useful for the purpose of this paper.

### B. Dose‐area product

The repeated measurements of an image by one observer with a densitometer gave a mean value of $393\pm 18\;{\text{Gy‐cm}}^{2}$. The mean value from different observers with a densitometer was $392\pm 21\;{\text{Gy‐cm}}^{2}$. Using the coefficient of variation as an indicator of variability, the intraobserver variability and interobserver variability of the manual technique are 4.5% and 5.3%, respectively (Table 1).

**Table 1:** Intraobserver variabilities and interobserver variabilities in five measurements of one image with a densitometer and five measurements of another image with a scanner

	DAP intraobserver variability		DAP interobserver variability	
Technique	DAP (Gy‐cm^2^)	Coefficient of variation	DAP (Gy‐cm^2^)	Coefficient of variation
Densitometer	393.2±17.8	4.5%	391.6±20.9	5.3%
Scanner	91.0±0.4	0.4%	92.6±1.7	1.8%

The repeated scans of a film in a fixed location in the scanner gave a mean value of $91\pm 1\;{\text{Gy‐cm}}^{2}$. When the film was moved to a different location for each scan, the mean value became $93\pm 2\;{\text{Gy‐cm}}^{2}$. The intraobserver variability and interobserver variability of the automatic technique with the scanner are 0.4% and 1.8%, respectively.

Among the 10 images used in the variability study, only one had a few (4 to be exact) aberrant pixels in the area of interest before the image was filtered as described earlier. Therefore, this defect could not be attributed to the film or to any bad detector elements in the scanner. The cause of this transient phenomenon remains unknown.

**Figure 7:**
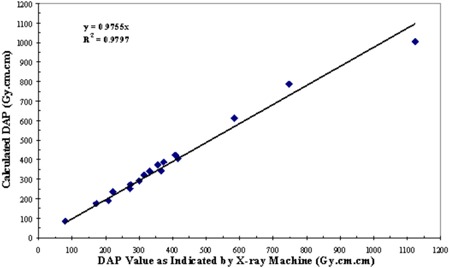
Results of DAP calculations from the densitometer. Solid line represents best fit by linear regression analysis.

**Figure 8:**
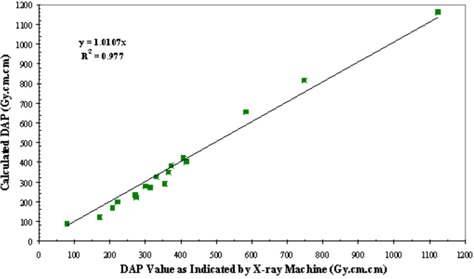
Results of DAP calculations from the scanner. Solid line represents best fit by linear regression analysis.

Dose–area product values from the manual technique with the densitometer are compared to the DAP values indicated by the machine in Fig. 7. The discrepancy has a minimum of 0.6% and a maximum of 10.6%. Regression analysis gives a slope of 0.98 and an $R^{2}$ value of 0.98. Dose–area product values from the automatic technique are compared with DAP values indicated by the machine in Fig. 8. The discrepancy has a minimum of 1.4% and a maximum of 19.8% difference from all these cases. Regression analysis gives a slope of 1.01 with an $R^{2}$ value of 0.98.

## IV. DISCUSSION

**Figure 9:**
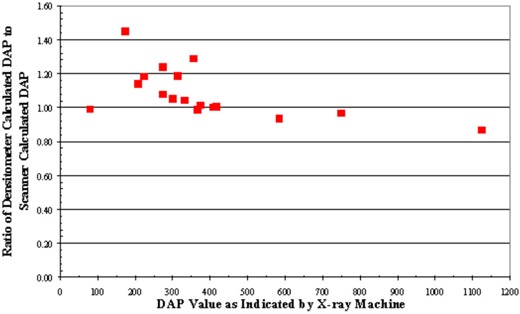
Ratio of densitometer‐calculated DAP to scanner‐calculated DAP versus the indicated DAP value by the X‐ray machine.

With a densitometer, the GafChromic XR Type R film has a usable range to about 14 Gy. With a scanner, the useable range is only about 6 Gy. These demonstrated upper limits were reached when the instrument was set to respond to the most sensitive spectral component of the film.^(^ ^8^ ^,^ ^9^ ^)^ The dynamic range might be extended by measuring the green color component instead. Using a properly calibrated densitometer, the derived DAP value from the filter deviated from the machine‐indicated value by less than 10%. A regression analysis was conducted to compare these two techniques. Regression analysis gives a slope of 0.98 and an $R^{2}$ value of 0.98. One‐tailed and paired *t*‐test yielded a *p*‐value of 0.38. The manual technique of outlining the radiation distribution patterns does introduce an experimental error of about 5%. This could account for the dispersion of the data about the fitted straight line in Fig. 7. Using a properly calibrated flatbed scanner with the appropriate calibration pattern, the derived DAP value from the film deviated from the machine‐indicated value by less than 20%. A regression analysis to compare these two techniques gives a slope of 1.01 with an $R^{2}$ value of 0.98. Two‐tailed and paired *t*‐test yielded a *p*‐value of 0.33. This automatic technique reduces the experimental error or precision to 0.4% and 1.8%, respectively. A direct comparison of the DAP obtained with the densitometer with the DAP obtained with the scanner shows an agreement within 20% (Fig. 9). Two‐tailed and paired *t*‐test yielded a *p*‐value of 0.76. An examination of Figs. 7 and 8 would show that in the few cases of significant discrepancies (>10%), the results from the two techniques deviate from the machine‐recorded value in the opposite directions.

The DAP values displayed by the X‐ray machine were calibrated with a reference thimble ion chamber. The reference scale in the densitometry measurements with the film had also been calibrated with the same ion chamber. Therefore, the DAP values derived from film recording and the DAP values derived more directly from the X‐ray machine have an uncertainty attributed to the accuracy error in calibration of the thimble ion chamber. The former technique could have additional uncertainties attributed to the mathematical model fitting to reference patterns (Figs. 2 and 6) and the precision in image processing. The piecewise polynomial fitting in Fig. 6 would give negligible error. While the X‐ray machine‐displayed DAP values are convenient and are readily available in newer equipment, they do not constitute absolute reference values. These are subject to other types of errors.^(^ ^11^ ^)^

## V. CONCLUSION

The limitations and accuracies in using GafChromic XR Type R film as an alternative means to determine DAP were studied in this investigation. While the use of the densitometer is more labor‐intensive and less precise (approximately 5% versus 2%), it can handle a wider range of radiation exposures (14 Gy versus 6 Gy). However, the range of the scanner could be extended if the green component of the image were used. The use of the flatbed scanner requires more initial investment in equipment and software, but the automatic technique is more precise and can be more efficient with further development in computer programming. Dose‐area product values derived from the film recording correlate very well with the X‐ray machine‐displayed DAP values. The slopes of the fitted linear relations in Figs. 7 and 8 suggest an overall agreement of the two techniques.
